# Effects of physical activity and melatonin on brain‐derived neurotrophic factor and cytokine expression in the cerebellum of high‐fat diet‐fed rats

**DOI:** 10.1002/npr2.12125

**Published:** 2020-07-18

**Authors:** Ai Sugiyama, Hisashi Kato, Hisashi Takakura, Seita Osawa, Yuki Maeda, Tetsuya Izawa

**Affiliations:** ^1^ Graduate School of Health and Sports Science Doshisha University Kyotanabe City Japan

**Keywords:** brain‐derived neurotrophic factor, cerebellum, cytokines, exercise training, high‐fat diet, melatonin

## Abstract

**Aims:**

Obesity suppresses brain‐derived neurotrophic factor (BDNF) expression and increases the expression of pro‐inflammatory cytokines. Herein, we assessed whether exercise training (ET), melatonin administration (MT), or their combination can affect the expressions of BDNF and cytokines in the cerebellum of high‐fat diet (HFD)‐fed rats.

**Methods:**

Wistar rats (4 weeks old) were divided into five groups: normal diet (ND)‐fed control (ND‐SED), HFD‐fed control (HFD‐SED), HFD‐fed ET (HFD‐ET), HFD‐fed MT (HFD‐MT), and HFD‐fed MT plus ET (HFD‐ETMT) group. The rats were fed ND or HFD for 17 weeks. Rats were subjected to ET (running on a treadmill) and/or MT (melatonin 5 mg/kg body weight, i.p.) for 9 weeks, 8 weeks after beginning the diet intervention. Changes in BDNF and cytokine expression levels were determined using immunoblotting and cytokine arrays, respectively, 36 hours following the last bout of ET.

**Results:**

Neither HFD‐ET nor HFD‐MT rats exhibited enhanced BDNF expression in the cerebellum, but HFD‐ETMT rats had higher level of BDNF expression compared with the others. The expression of TrkB, a BDNF receptor, was higher in HFD‐ETMT rats than in HFD‐ET and HFD‐MT rats. HFD enhanced the expression of interleukin (IL)‐1, IL‐2, and interferon‐γ but reduced the expression of IL‐4, IL‐6, and IL13. ET and ET plus MT counteracted these HFD‐induced changes in cytokine expressions.

**Conclusion:**

Exercise in combination with melatonin confers the potential benefits of increasing BDNF and improving HFD‐induced dysregulations of cytokines in the cerebellum.

## Abstract 2

High‐fat diet feeding enhanced the expressions of pro‐inflammatory cytokines but reduced those of anti‐inflammatory cytokines in the rat cerebellum. Interestingly, exercise training and exercise in combination with melatonin administration had a reversal effect on high‐fat diet feeding‐induced changes in cytokine expressions.

## Graphical abstract

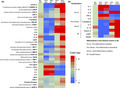

## 1 INTRODUCTION

Recent studies suggest that obesity and high‐fat diet (HFD) feeding with peripheral inflammation lead to deterioration in cognitive function and neurogenesis, probably via both, the dysregulation of brain‐derived neurotrophic factor (BDNF) and the increase in brain inflammation.^1^, ^2^, ^3^ Therefore, it is important to verify the effects of behavior and pharmacological interventions for improving obesity on both, brain BDNF and inflammation levels.

Interventions such as exercise training (ET) and melatonin administration (MT) have been shown to increase BDNF levels in the mouse hippocampus,^4^, ^5^, ^6^ and MT reportedly potentiates ET‐induced neurogenesis in the rodent hippocampus.^7^ It can, therefore, be postulated that the combinatorial effect of ET and MT to enhance brain BDNF levels is greater than that of their individual effects. However, a verification is warranted on whether a combination of ET and MT can enhance BDMF level in brain region(s) besides the hippocampus in obesity. Moreover, there is little evidence on whether ET or MT, or both combined, can ameliorate HFD‐induced brain inflammation.

The cerebellum is one of regions directly engaged in locomotor control, and it has recently been shown that a 3‐week running exercise regimen brought the experimental groups' depression‐associated low cerebellar BDNF levels on par with the control group's.^8^ Furthermore, improvement of cerebellar inflammation has been shown to revert inflammation‐induced depression‐like behaviors.^9^ Based on the above, we selected the cerebellum and hypothesized that ET combined with MT may be an efficacious intervention against obesity‐related changes in cerebellar BDNF and inflammation levels. To this end, we assessed whether ET, MT, or a combination of both, could affect the expression of BDNF, its tyrosine kinase receptor B (TrkB), and cytokines in the cerebellum of HFD‐induced obese rats.

## 2 METHODS

### 2.1 Animals and intervention program

Male Wistar rats (4 weeks old: SLC) were housed in a room at 23°C with a 12:12‐hour light‐dark cycle. All animals were divided into five groups (four rats in each group): normal diet (ND)‐fed sedentary (ND‐SED), HFD‐fed sedentary (HFD‐SED), HFD‐fed ET (HFD‐ET), HFD‐fed MT (HFD‐MT), and HFD‐fed ET plus MT (HFD‐ETMT) group. ND‐SED rats were fed a standard diet (MF, Oriental Yeast), and the rats in the HFD group were fed HFD (60% fat, Research Diets) for 17 weeks. Water and food were available ad libitum.

Exercise training and MT were started 8 weeks after the beginning of dietary intervention. HFD‐ET and HFD‐ETMT rats ran on a treadmill (5‐degree incline), 5 d/wk, for 9 weeks according to a protocol reported.^10^ The running time and speed were increased progressively until after 6 weeks, when the rats ran continuously for 90 minutes at 30 m/min. HFD‐MT and HFD‐ETMT rats received an intraperitoneal injection of MT at 5 mg/kg body weight for 9 weeks. The dose of melatonin administered was based on previous studies.^7^, ^11^ Following all interventions, the rats were euthanized with pentobarbital sodium (0.5 mg/kg body weight, i.p.; Kyoritsu Seiyaku), and the cerebellum was removed. HFD‐ET and HFD‐ETMT rats were euthanized at least 36 hours after the last exercise session. All experiments were approved by the Animal‐Care Committee of Doshisha University.

### 2.2 Immunoblotting analysis

The cerebellum was homogenized in ice‐cold EzRIPA lysis buffer (ATTO). The homogenate was centrifuged twice for 20 minutes at 14 000 *g* at 4°C; the total protein concentration in the supernatant obtained was then measured using a BCA protein assay kit (Takara Bio). The same amounts of protein in each sample were run on SDS‐PAGE (8%‐12.5% gel). After electrophoresis, the proteins were transferred onto a PVDF membrane and blocked for 5 minutes in Bullet Blocking One (Nacalai Tesque) or for 60 minutes with Tris‐buffered saline (20 mmol/L Tris, 0.15 mol/L NaCl, pH 7.4) containing 0.1% Tween‐20 and 5% skimmed milk. Membranes were incubated overnight at 4°C with a 1:1000 dilution of specific antibodies: BDNF, TrkB, GAPDH (Abcam); cAMP response element binding protein (CREB) and phospho‐CREB (CST Japan). The membranes were labeled for 60 minutes with anti‐rabbit or anti‐mouse immunoglobulin G (1:2500; GE Healthcare). Bands were visualized using the ECL system (GE Healthcare) and quantified on the ChemiDocTM MP system (Bio‐Rad). Protein abundance was normalized to GAPDH.

### 2.3 Cytokine array analysis

Cytokine array analysis was performed using the Rat Cytokine Antibody Array (Abcam) according to the manufacturer instruction. Protein samples from individual rats were pooled to ensure equal volumes in each experimental group. This compensated for lower volume samples and could mitigate the effects of biological sample variation. Images were acquired using the ChemiDocTM MP system (Bio‐Rad), and the pixel intensity was quantified using Image J (National Institutes of Health).

### 2.4 Statistical analysis

All data, except the cytokine array analysis data, are presented as means ± SE and were analyzed by one‐way analysis of variance. Where applicable, the Bonferroni test for multiple comparisons was conducted. A *P*‐value < .05 or less following post hoc analysis was considered significant. All analyses were performed using the Excel software package.

## 3 RESULTS

The final mean body weight (g) was significantly lower in HFD‐MT (344.3 ± 5.2) (*P* < .01) and HFD‐ETMT (329.3 ± 4.8) (*P* < .001) than in HFD‐SED (404.0 ± 12.5) rats; no significant difference was found between HFD‐MT and HFD‐ETMT groups. While the mean body weight in HFD‐ET (368.0 ± 14.1) rats was lower than in HFD‐SED rats, no statistical significance was found (*P* = .162).

**FIGURE 1:**
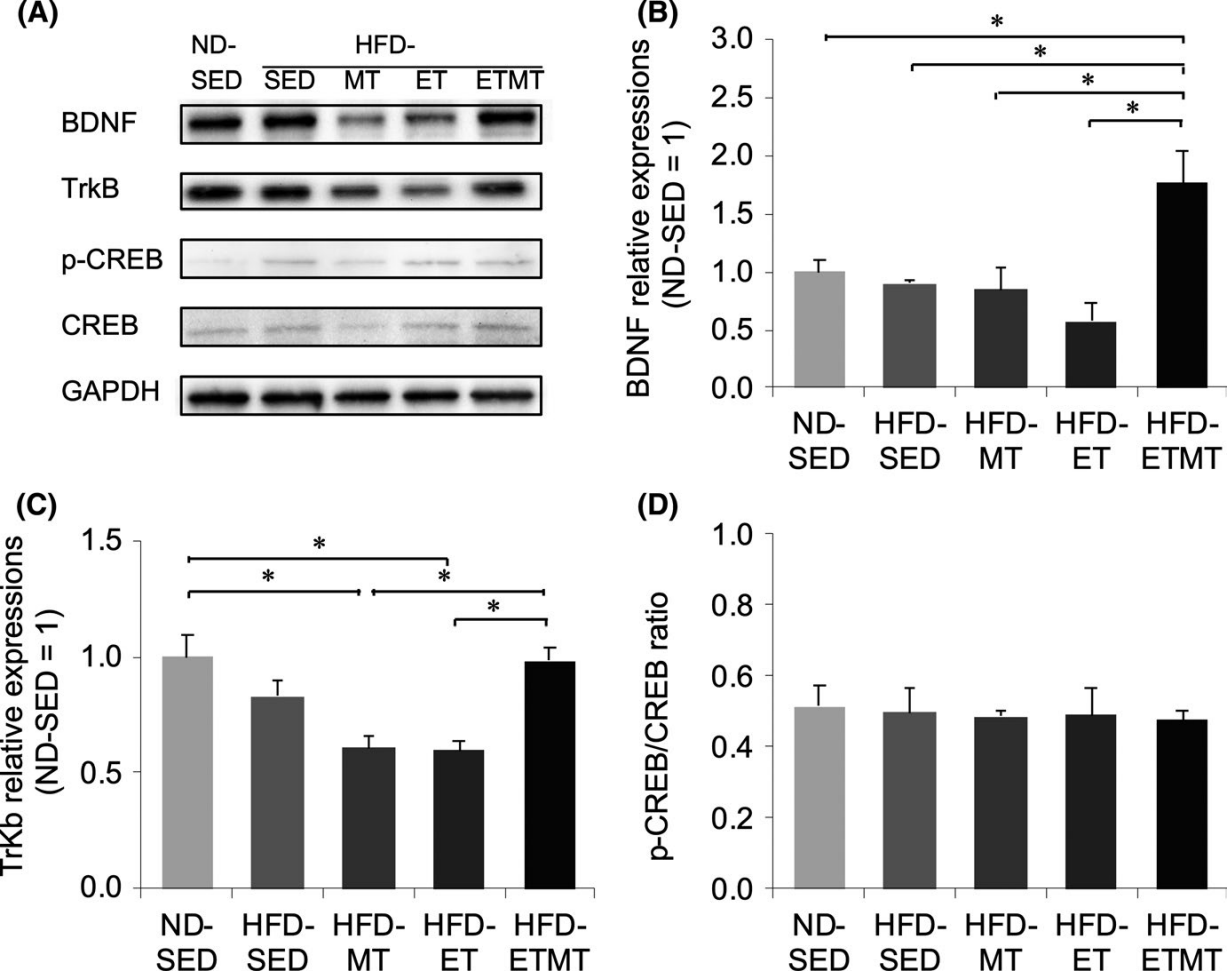
The level of protein expression in the cerebellum following 9 wk of intervention. A, Representative band of BDNF, TrKB, CREB, phosphor‐CREB (p‐CREB), and GAPDH. Band intensities of (B) BDNF and (C) TrkB were normalized to those of GAPDH, and the value is expressed in relation to the value of HFD‐SED rats (set to 1). D, p‐CREB/CREB ratio. Data are presented as the mean ± SE (n = 4, each). *P < .05

The cerebellar BDNF expression was not significantly different between ND‐SED, HFD‐SED, HFD‐MT, and HFD‐ET rats (Figure 1B), but HFD‐ETMT rats exhibited a higher expression of BDNF than HFD‐SED (*P* < .05), HFD‐MT (*P* < .05), and HFD‐ET rats (*P* < .01). The expression of TrkB, a receptor for BDNF, was lower in HFD‐MT (*P* < .01) and HFD‐ET (*P* < .01) than in HFD‐ETMT rats, but that was not significantly different among HFD‐SED, HFD‐MT, and HFD‐ET rats (Figure 1C). No significant difference was found in phospho‐CREB/CREB ratios among the groups (Figure 1D).

**FIGURE 2:**
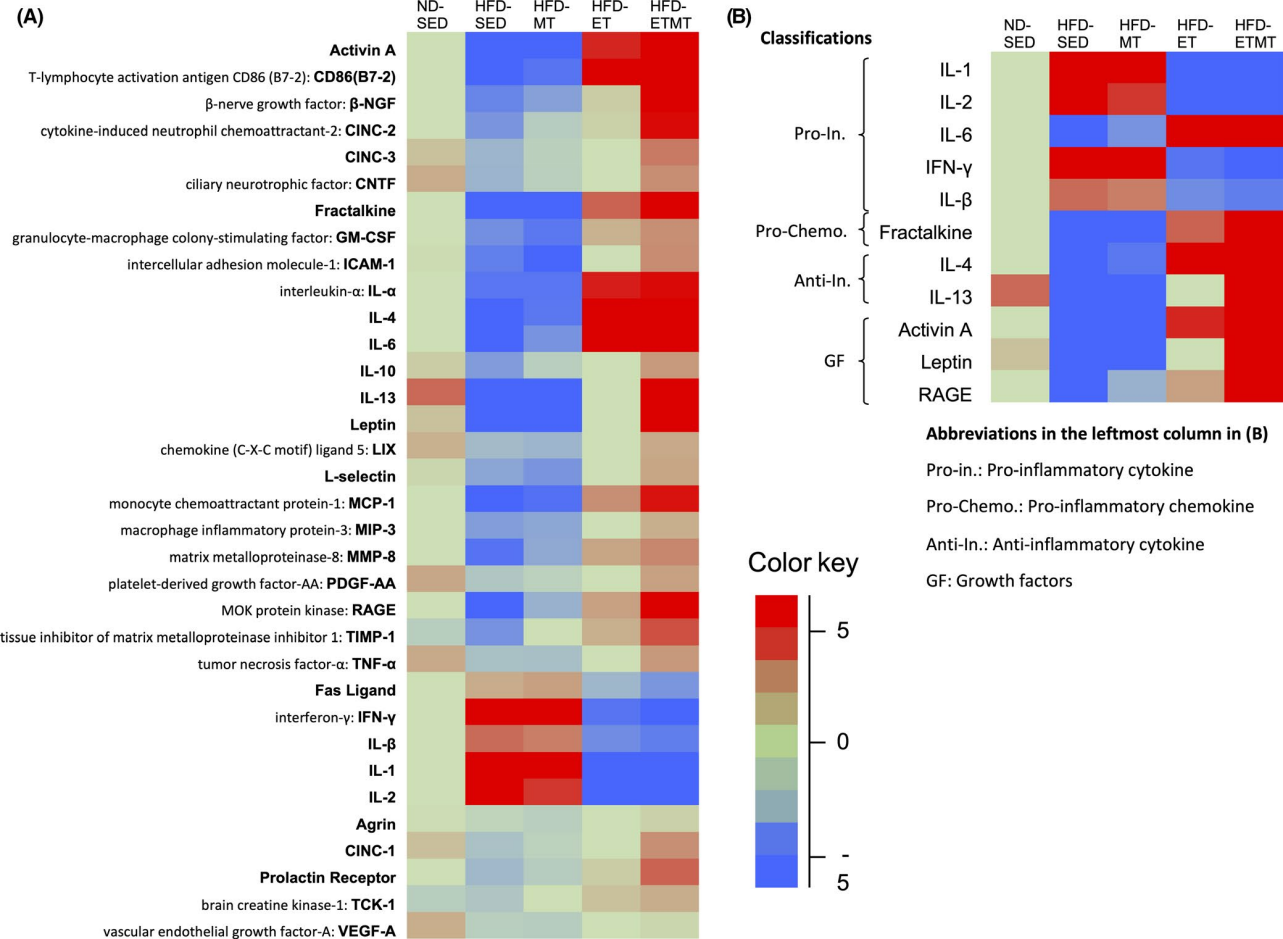
Heat map of cytokine expression in the cerebellum following 9 wk of intervention. The relative expression levels (fold change) of each cytokine were determined by comparing the designated protein concentration of each sample relative to the median value of the designated cytokine across all samples. A, Heat map for all 34 cytokines, B, The cytokines displaying greater than twofold changes were selected in HFD‐SED rats. The images were shown in Figure S1. The color red indicates relatively high protein expression, and the color blue indicates relatively low protein expression

Figure 2 shows the cytokine expressions profiles. The heat map in Figure 2B demonstrates that HFD caused at least a twofold downregulation of seven cytokines, and a twofold upregulation of four cytokines; HFD enhanced the protein levels of pro‐inflammatory cytokines, interleukin (IL)‐1, IL‐2, IL‐lβ, and IFN‐γ but reduced those of anti‐inflammatory cytokines, IL‐4, and IL‐13. Interestingly, ET contracted HFD‐induced changes in cytokine expression, and MT had a tendency to enhance the contracted effect of ET.

## 4 DISCUSSION

Recent advances suggest that obesity and HFD feeding deteriorates cognitive function via abnormalities of BDNF levels.^1^, ^2^, ^3^ However, this report did not observe HFD‐induced reduction of cerebellar BDNF. It appears valid to conclude that the effect of HFD on brain BDNF levels remains unclear; some studies reported that HFD reduced BDNF levels in the hippocampus^12^, ^13^, ^14^ and cerebral cortex^15^ but had no effect on hippocampal BDNF.^16^, ^17^ Furthermore, HFD‐ET and HFD‐MT rats did not exhibit enhanced cerebellar BDNF expression. In this regard, a site‐specific difference of BDNF expression may exist between the hippocampus and cerebellum in response to ET. Second, the effect of ET on BDNF expression may be considered as an acute effect rather than a chronic one^18^; the increased hippocampal BDNF expression was found at 2 hours, but not 2 days after 4 weeks of ET.^5^ We collected the cerebellum 36 hours after the last exercise session. Finally, the doses of melatonin were probably inadequate for increasing BDNF expression; a report suggests that even at 40 mg/kg for 21 days, melatonin increased hippocampal BDNF levels by only 17% compared with controls.^19^

Even under these conditions, ET combined with MT demonstrated elevated BDNF expression 36 hours after the intervention. This effect is unlikely to be due to the intervention‐induced inhibition on body weight gain; ET and MT both significantly inhibited or tended to inhibit HFD‐induced body weight gain, respectively, similar to ET and MT combination. Thus, there may be a functional relationship between ET and MT.

Melatonin promotes BDNF production through at least CREB phosphorylation, by extracellular‐signal‐regulated kinase activation.^12^, ^20^ BDNF‐occupied TrkB stimulates the mitogen‐activated protein kinase (MAPK)/phosphatidylinositol‐3‐kinase (PI3K)/phospholipase Cγ (PLC) pathway, resulting in de novo expression of *Bdnf* gene.^3^, ^21^ Exercise also activates CREB through the cAMP‐dependent pathway.^22^ It was therefore expected that ET and MT in combination would additively enhance BDNF expression through their own signaling pathways. However, no significant difference was found in phospho‐CREB/CREB ratios among the groups. This finding may contradict the increased expression of BDNF and TrkB in HFD‐MTET rats and lower expression of TrkB in HFD‐MT and HFD‐ET rats, compared with HFD‐SED and HFD‐ETMT rats. The translation and transcription of BDNF are regulated by multiple signaling cascades.^3^ Therefore, further studies are required to explore the effects of ET, MT or their combination on other pathways driving expression/function of BDNF, such as the MAPK/PI3K/PLC signaling pathway.^3^ A possible cause for lower expression of TrkB in HFD‐MT and HFD‐ET rats also remains to be established.

Obesity‐ and HFD feeding‐induced systemic inflammation causes central inflammation.^1^, ^2^, ^3^ Our heat map shows that HFD elevated some inflammatory cytokines and decreased some anti‐inflammatory cytokines in the cerebellum, while showing that both, ET alone and in combination with MT counteracted such HFD‐induced changes in inflammatory cytokine expression. The most likely reason for this counteracted effect of ET may be its ability to suppress adipose tissue inflammation^23^ and microglial activation.^24^ HFD‐induced adipose inflammation increases circulating pro‐inflammatory cytokines, which in turn activate microglia, the brain immune cells.^1^ Microglial activation accelerates brain inflammation, and depleting microglia abrogates HFD‐induced inflammation.^25^ Thus, ET‐suppressed adipose inflammation^23^ and microglial activation^24^ may improve HFD‐induced cerebellar inflammation. The antioxidant property of ET may also be responsible. Oxidative stress promotes pro‐inflammatory cytokine production, and both inflammation and oxidative stress often coexist.^1^ ET can suppress the production of cerebellar oxidative stress markers, malondialdehyde,^26^ and thiobarbituric acid reactive substances.^27^ Melatonin may potentiate the effect of ET through its antioxidant and anti‐inflammatory properties.^28^

Brain‐derived neurotrophic factor and inflammatory cytokines are believed to affect the expression/function of each other; BDNF downregulates TNF‐α expression and upregulates IL‐10 expression^29^; IL‐1β upregulates or decreases both, hippocampal BDNF and TrkB expression following single or chronic injections, respectively.^30^ However, such an orchestrated change in BDNF and inflammatory cytokine expression was not always found; although the expression profiles of cytokines were quite different between all groups, elevated BDNF levels were found in HFD‐MTET rats only. More detailed data will be needed before establishing the functional interaction of BDNF expression with inflammatory cytokines.

In conclusion, our data suggest that ET combined with MT may play a potential role in elevating BDNF and improving HFD‐induced dysregulations of cerebellar cytokines.

## CONFLICT OF INTEREST

The authors declare no conflict of interest.

## AUTHOR CONTRIBUTIONS

AI, HT, and TI conceived and designed the study. AI performed most of the experiments with assistance from HK, SO, YM, and HT. AI and TI analyzed the data and wrote the manuscript. TI edited the manuscript.

## ANIMAL STUDIES

All animal experiments were approved by the Animal‐Care Committee of Doshisha University.

## Supporting information

Figure S1

Data S1

## Data Availability

The data that support the findings of this study are available in the Data S1 of this article.
