# Supplementary material for: Effects of physical activity and melatonin on brain‐derived neurotrophic factor and cytokine expression in the cerebellum of high‐fat diet‐fed rats
Source: Neuropsychopharmacol Rep. 2020 Jul 18;40(3):291–6. doi: 10.1002/npr2.12125 (PMC7722650; doi:10.1002/npr2.12125)
Supplement: Supplementary file 1 — Figure S1 [file NPR2-40-291-s001.docx]

Supplementary Figure S1


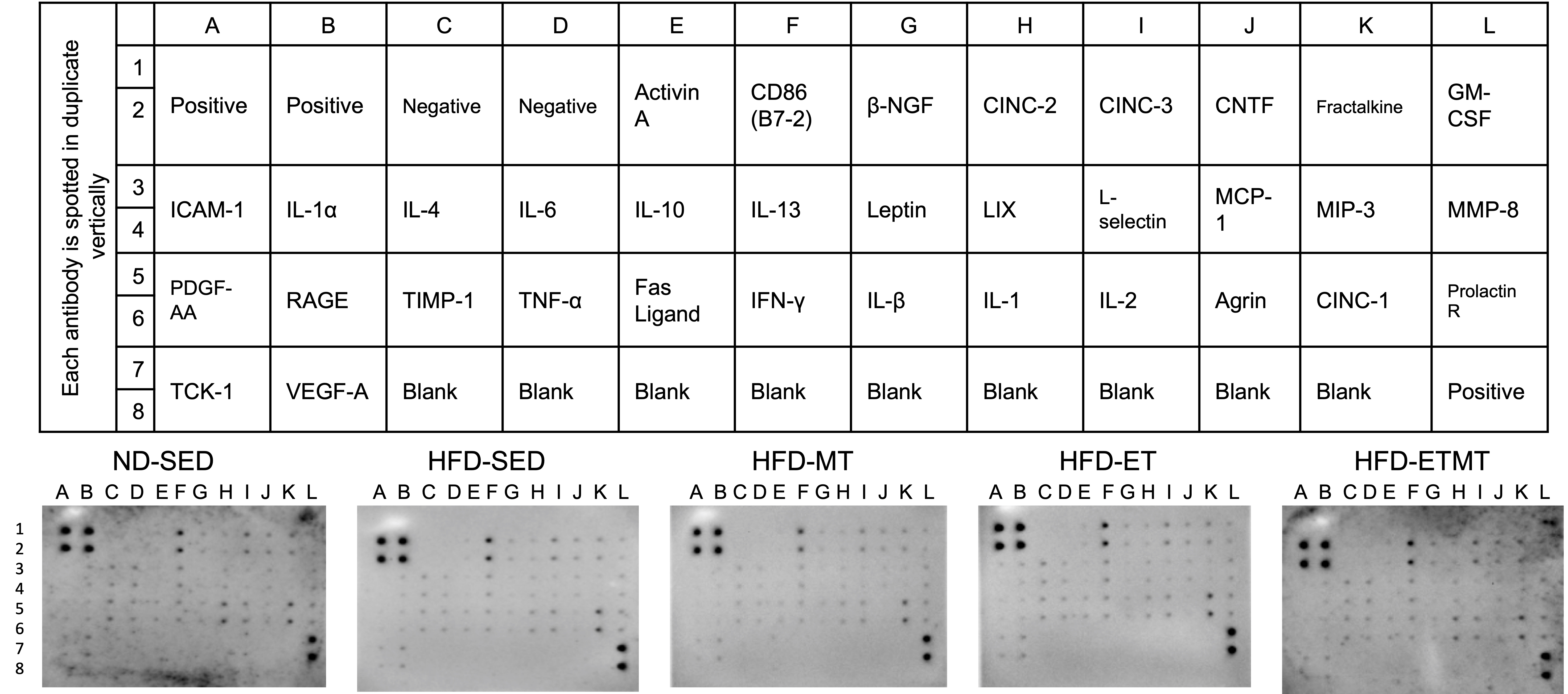


Figure S1. (A) The alignment of 34 cytokines on Rat Cytokine Antibody Array (Membrane, 34 targets) (Abcam). Abbreviations. POS, positive spots; NEG, negative spots. Other abbreviations were described in Fig. 2. (B) Representative images of cytokine array blots. Each blot represents immunoreactive staining against respective antibodies, and the staining at the negative control and blank slots was absent. The relative expression levels of each cytokine were determined by comparing the pixel intensity of the respective blots to that of the positive control on the same array. ND-SED, normal diet-fed sedentary; HFD-SED, high-fat diet-fed sedentary; HFD-MT, HFD-fed melatonin administration (MT); HFD-ET, HFD-fed exercise trained (ET); HFD-ETMT, HFD-fed ET plus MT group.
